# Developing Digital Tools for Remote Clinical Research: How to Evaluate the Validity and Practicality of Active Assessments in Field Settings

**DOI:** 10.2196/26004

**Published:** 2021-06-18

**Authors:** Jennifer Ferrar, Gareth J Griffith, Caroline Skirrow, Nathan Cashdollar, Nick Taptiklis, James Dobson, Fiona Cree, Francesca K Cormack, Jennifer H Barnett, Marcus R Munafò

**Affiliations:** 1 School of Psychological Science Faculty of Life Sciences University of Bristol Bristol United Kingdom; 2 Medical Research Council Integrative Epidemiology Unit University of Bristol Bristol United Kingdom; 3 Population Health Sciences Bristol Medical School University of Bristol Bristol United Kingdom; 4 Cambridge Cognition Ltd Cambridge United Kingdom; 5 Cambridge Cognition Ltd Cambridge, MA United States; 6 Department of Psychiatry University of Cambridge Cambridge United Kingdom

**Keywords:** digital assessment, remote research, measurement validity, clinical outcomes, ecological momentary assessment, mobile phone

## Abstract

The ability of remote research tools to collect granular, high-frequency data on symptoms and digital biomarkers is an important strength because it circumvents many limitations of traditional clinical trials and improves the ability to capture clinically relevant data. This approach allows researchers to capture more robust baselines and derive novel phenotypes for improved precision in diagnosis and accuracy in outcomes. The process for developing these tools however is complex because data need to be collected at a frequency that is meaningful but not burdensome for the participant or patient. Furthermore, traditional techniques, which rely on fixed conditions to validate assessments, may be inappropriate for validating tools that are designed to capture data under flexible conditions. This paper discusses the process for determining whether a digital assessment is suitable for remote research and offers suggestions on how to validate these novel tools.

## Introduction

The emergence of SARS-CoV-2 at the turn of 2020 demonstrates how abruptly life—and research—can change. The global response to the resulting pandemic also demonstrates how quickly the world can use technology to adapt to these changes. The physical closure of organizations has less impact now than it would have had even 10 years ago; thanks to technological advances, many formerly in-person activities can now be conducted virtually. For some organizations, this way of operating was already familiar, whereas for others it is novel and challenging. Overall, most organizations are being compelled to adapt and create innovative ways to enhance remote working.

Scientific research has also had to adapt to these unforeseen circumstances. Fortunately, a great deal of psychological research was routinely conducted remotely before the SARS-CoV-2 outbreak [1], primarily in an attempt to produce more externally valid research [2,3]. Remote data collection offers an opportunity for researchers to broaden the diversity of their samples, both in terms of whom they recruit and when and where data collection occurs. Remote data collection is facilitated through web-based recruitment platforms (eg, *Amazon Mechanical Turk*, *Prolific*, and *Call for Participants*), web-based survey and experiment builders (eg, *Qualtrics* and *Gorilla*), and personal devices (eg, smartphones and smartwatches) that can be used to collect data on a range of behaviors (eg, location, movement, social interactions, travel behavior, energy intake, energy expenditure, vital signs, sleep patterns, menstrual cycles, mood, cognition, and pain) [1,3,4]. Smart devices are used for both active and passive data collection, and users can manually input self-report data, whereas built-in sensors allow for continuous collection of objective data [3].

Although much cognitive and behavioral research was already moving toward remote testing before the SARS-CoV-2 outbreak, progress in this field needs to be accelerated. Once social distancing measures are relaxed, it is reasonable to expect a gradual return to normality. However, it is unrealistic to expect our way of life to be wholly unchanged. With the world turning to technologies that facilitate virtual interactions, there are likely to be technical improvements made to these tools, as well as increased availability. The discovery that certain virtual experiences are equally efficient as, or more efficient than, their real-world counterparts may change the way that many of us operate. Considering the widespread effects of the current pandemic and the potential for similar infectious disease pandemics in the future, it is realistic to expect that virtual research will become increasingly popular and, perhaps, even the new norm [5]. Now, more than ever, resources need to be invested in the development of remote research assessments.

**Figure 1:**
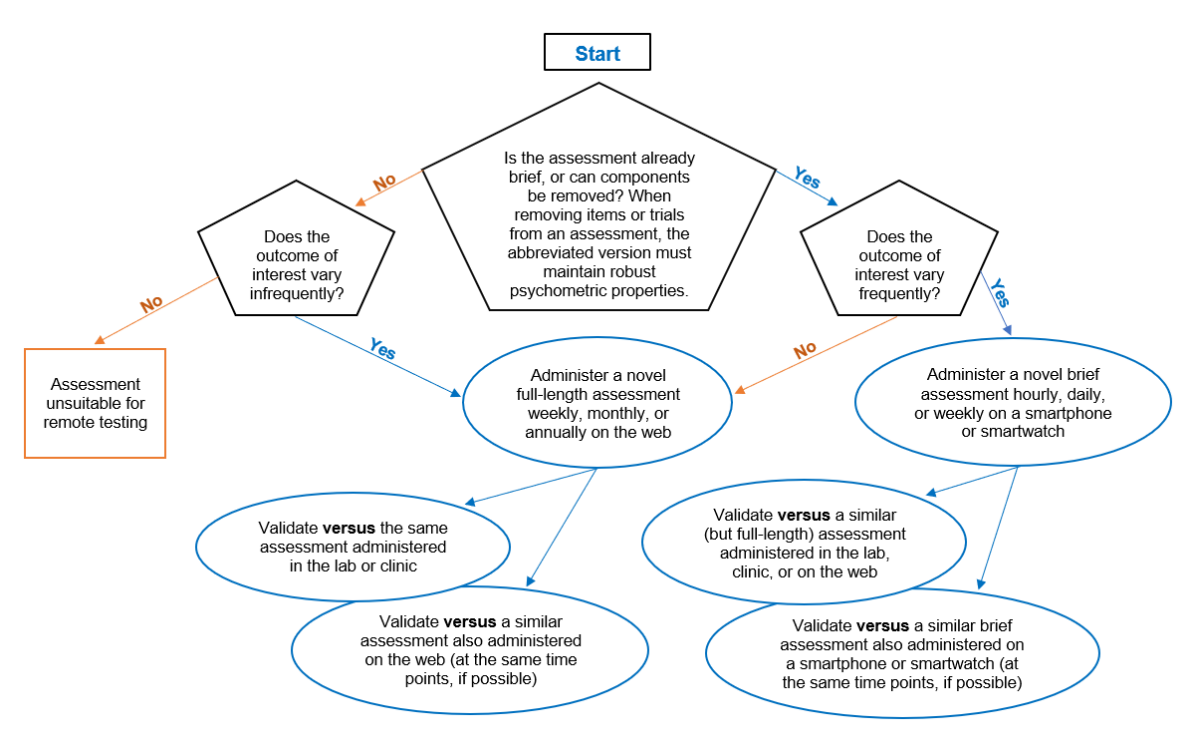
The decision process for validating digital assessments for remote research.

Here, we discuss the benefits of conducting remote clinical research, how to determine the suitability of an assessment for remote research, and various approaches to validating such assessments based on where and how frequently data collection occurs. We focus on the validation process of active assessments, including both objective and subjective measures (neuropsychological tests and patient-reported outcomes, respectively). However, the principles outlined here should also apply to the validation process of some passive assessments, such as those designed to detect cigarette smoking [6]. We have included a flowchart (Figure 1) to illustrate the decision-making process. We discuss traditional validation techniques (ie, the comparison of a single assessment between controlled and uncontrolled settings and comparison between two different assessments in the same setting), as well as innovative methods that account for the measurement of constructs across time and location (eg, improving signal-to-noise detection to capture more robust baselines and develop novel phenotypes for improved precision in diagnosis and accuracy in outcomes). We have included case studies to illustrate the breadth of approaches and techniques that may be necessary to consider when designing a validation process for novel assessments. However, it is important to note that these individual pilot studies are only presented here for example purposes and should not be considered comprehensive empirical studies in their entirety.

## The Benefits of Conducting Clinical Research Remotely

In a conventional clinical trial, researchers ask patients to complete comprehensive assessments to monitor their symptoms. The assessments can be extensive and may require trained personnel to administer and score them. As the assessments are burdensome for both parties [7], the frequency with which they can be administered is limited and, as a result, they only provide snapshots of treatment efficacy. In other words, assessments may only be administered monthly or even less frequently. However, symptoms can fluctuate from week to week, day to day, and even within a single day. At the time of assessment, a patient’s symptoms might improve or be exacerbated by chance because of factors unrelated to treatment efficacy (eg, a stressful event occurring in the morning before an afternoon assessment). Therefore, it can be difficult to ascertain whether changes in patients’ symptoms are due to treatment or extraneous factors. Researchers might test patients on the same day of the week or at the same time of the day throughout the trial to account for fluctuations in symptoms. However, this strategy operates on the assumption that symptom changes occur in a predictable manner.

Many psychiatric disorders are characterized by irregular circadian rhythms. Therefore, these patient groups might have, in particular, stochastic fluctuations in cognitive function and mood that cannot be easily predicted [8]. Researchers may attempt to evaluate symptoms during the intervals between assessments by relying on retrospective subjective reports from patients [3]. However, self-report measures can be unreliable indications of actual behavior [9-12], especially when a patient’s condition affects their insight or their memory [13,14], impairing their ability to accurately recall past events and symptoms. In addition, there is a limit to how much detail can be recalled. Retrospective subjective reports usually ask patients to reflect on symptom changes over a week or month because recalling hourly or daily changes would be unfeasible [15]. To account for confounders that coincide with test days and avoid relying on biased reports of symptoms, researchers may measure the symptoms of interest before and after an experimental manipulation in the laboratory. This design is assumed to act as a proxy for what might occur in the real world. However, it is not clear how well the trigger and resulting behavior in an artificial setting will translate into those occurring in the real world [16]. An alternative is to conduct the trial remotely, using a phone or wearable device to administer assessments in natural settings at regular intervals or in response to state changes, also known as ecological momentary assessment [2,3]. This methodology reduces user burden while increasing the likelihood of the measurements capturing clinically relevant symptoms when they occur in real time, and such approaches will help to revolutionize clinical trials [3,17].

## Determining the Suitability of an Assessment for Remote Research

When developing any assessment tool, there needs to be a trade-off between the length or duration of an assessment and the frequency at which it can be administered. On the one hand, the testing time should be kept to a minimum. Increased testing time can cause participants to tire of the assessments [18], which could decrease the accuracy of responses and compliance, thereby increasing attrition. Similarly, knowing that one will need to complete frequent lengthy assessments during a research study may negatively affect recruitment to that study. On the other hand, there needs to be a sufficient amount of data to maximize the precision of the measurements and ensure their accuracy (ie, effectiveness at detecting the presence or absence of the symptom of interest) [19]. These considerations are especially crucial when the research study requires participants to interrupt their normal routines to complete assessments [20,21] and even more so when conducting clinical research with patients who may have a low threshold for burdensome research procedures because of their symptoms (eg, lack of concentration, fatigue, or motivational fluctuations). Therefore, for the most part, remote assessments should be either *brief and frequent* or *lengthy and infrequent* to be successfully implemented and to reliably capture valid data. However, there are exceptions to this rule, which we discuss herein.

Assessments that require a substantial amount of time to complete cannot be easily incorporated into daily routines or administered frequently without overly inconveniencing users. The inclusion of these assessments in real-world trials is likely to decrease compliance and increase attrition. Therefore, lengthy assessments will need to be abbreviated to be administered at high frequencies.

However, some assessments cannot be abbreviated, such as those that cannot sacrifice items or trials without degrading the assessment’s psychometric properties. As long as lengthy assessments can be administered at relatively low frequencies (eg, once a day or once a week) and have some flexibility regarding when they can be completed (within reason), they can be administered remotely (ie, on the web). Administering a lengthy assessment remotely (as opposed to administering it at a testing facility) provides a more naturalistic context for data collection and may reduce confounding factors associated with artificial test settings. It also reduces the intervals between events of interest and subsequent measurements, which may improve symptom recall accuracy. In addition to these benefits, there are temporal and spatial limitations within this context that should be considered. Users will need to find an appropriate space as well as time to engage with the assessment. Therefore, administering lengthy assessments remotely is not a suitable method for capturing clinically relevant data in real time. However, depending on the research question and clinical population, it might be preferable for participants to complete an extensive assessment less frequently and retrospectively, rather than complete a less comprehensive assessment more frequently and in real time (eg, when qualitative data are needed, for the purposes of a clinical diagnostic interview, etc).

Assessments that are brief (or can be abbreviated), track dynamic changes, and are not limited by specific technical requirements are appropriate for high-frequency remote testing. This approach captures high-resolution data that allow for the interpretation of outcomes in relation to time and location. This increased level of detail can be incorporated into the statistical analyses to improve the signal-to-noise ratio. As a result, high-frequency assessments can be used to achieve more representative baselines and develop novel digital phenotypes to improve the precision and accuracy of diagnosis and outcomes [22]. It should be noted that brief assessments do not necessarily have to be administered at high frequencies to be valid measurement tools. For example, the 2-item Patient Health Questionnaire (PHQ) is an abbreviated version of the 9-item PHQ, which is widely used to evaluate depression and demonstrates sufficient diagnostic sensitivity when administered at the same low frequency as the full-length version [23]. Administering the abbreviated version infrequently is likely to be less sensitive than both administering the full-length version infrequently and administering the abbreviated version frequently. However, the latter options may not always be feasible (eg, when the other study procedures are already time consuming and effortful) or sensible (eg, when increasing the response rate or completion rate is key).

Sampling frequency matters—both over- and undersampling can have negative consequences. It may be inefficient to use hourly sampling to capture diurnally varying symptoms [24] or to use time-based sampling to capture symptoms that occur in response to specific (eg, clinically relevant) events [3]. This is particularly true if the frequency and regularity of the relevant event vary considerably among individuals (eg, panic attacks can occur several times per week or a couple of times per year) [25-27]. Sampling more than necessary risks burdening participants, wasting resources, and ultimately degrading data quality (eg, by decreasing compliance) [24]. The sampling frequency should align with the fluctuations of the symptoms of interest as much as possible so that each measurement is informative. This may mean that low-frequency sampling is the most appropriate, circumventing the need for brief assessments. However, compliance may be low if participants are required to self-initiate assessments after the occurrence of relevant events. Alternatively, high-frequency sampling can be used to continuously monitor relevant events (eg, through a watch that passively detects smoking) and trigger an assessment when appropriate [6,28,29]. There are also cases in which the relevant events may occur irregularly or infrequently, but continuous monitoring of the symptoms of interest outside of the event window is useful; for example, measuring positive and negative affect regularly as well as whenever self-harm occurs [30]. Continuous monitoring of symptoms provides a clearer picture of baseline functioning, which can be used to better characterize changes in functioning. Furthermore, continuous monitoring of symptoms of interest, relevant events, or related factors can help identify patterns in behavior that can be used to predict changes, increasing sampling accuracy and reducing participant burden [31,32]. Of course, the feasibility of continuous monitoring will depend on the effort required by the participant to complete the assessment and the capacities and constraints of the specific participant group.

There are also assessments that are appropriate to abbreviate and administer at high frequencies but may not be suitable for remote testing for other reasons (eg, if researcher supervision or a specialist device is required). Furthermore, if the assessment is susceptible to practice effects, mitigating solutions will need to be developed [33]. Some considerations may not be directly related to the assessment itself but to the context in which it is used. For example, an assessment may be designed to evaluate outcomes after a pharmacological challenge and, in this case, whether or not the pharmacological challenge can be delivered remotely needs to be considered. It may be possible, if appropriate precautions are taken, to instruct participants to self-administer certain substances, such as caffeine, alcohol, nicotine, etc, but this would clearly not be possible in other cases (eg, controlled substances).

## A Process for Evaluating Tools for Remote Research

As research transitions from operating in testing facilities to the field, it is vital that remote research assessments are developed to a high standard. A remote research assessment needs to be both a valid measure of the construct being evaluated and practical to implement. One of the difficulties in transitioning to remote data collection lies in defining the process for validating remote assessments. For a novel assessment to be valid, it must be reliable and a true measure of the construct of interest. Reliability can be verified by measuring the internal consistency of items or trials or by investigating whether the assessment produces consistent results under similar conditions (ie, test-retest reliability) [34]. To demonstrate internal validity, a reliable assessment is compared with a gold standard (ie, a tool that has been demonstrated to consistently and accurately measure the construct of interest) under controlled conditions to reduce extraneous influences. To demonstrate external validity or generalizability, the assessment is administered and compared across different testing conditions (ie, at different times, in different settings, and in different people) [35,36].

However, this paradigm for evaluating internal validity is not necessarily useful for validating remote research assessments, which are not designed to be administered under controlled conditions. This is not necessarily a limitation because using a traditional validation paradigm may not be ideal when the focus is on real-world behavior, as is the case in applied research. Testing under controlled conditions can introduce temporal and spatial biases into the data. Unlike traditional research assessment, remote research assessment is far more flexible in terms of when and where it can be administered. This increased flexibility in data collection can improve the external validity of the assessment but also means less standardization because assessments are completed without researcher supervision and in contexts that can vary within and across participants. Therefore, the framework for evaluating the *internal* validity of remote research assessments may need to be different from traditional methods that assume spatiotemporal consistency.

## How to Validate Low-Frequency Assessments for Remote Research

The internal validity of an assessment includes face, content, criterion, and construct validity [36,37]. Criterion validity is useful to assess when evaluating the construct validity of an assessment, abbreviating an already existing assessment, or planning to use an assessment in a new environment. To assess the criterion validity of any new assessment (either a completely new assessment or an amended version of an already validated assessment), the validity assessment needs to be administered (concurrent validity) or after (predictive validity) an established assessment. An established assessment is one that has already been validated to measure the same construct or a similar constructs (to evaluate criterion and construct validity, respectively) [36]. The outcomes generated by the new assessment need to be compared with those generated by an established assessment. To validate a new low-frequency assessment for data collection in a clinical or laboratory setting, both the new and established assessments need to be administered under standardized conditions at the testing facility to confirm that the assessments are equivalent.

To validate a new low-frequency assessment for remote data collection, the new assessment needs to be administered remotely and the resulting outcomes compared with those generated by the established assessment. The established assessment can either be administered in a clinical or laboratory setting or remotely (depending on whether the assessment has already been validated for remote data collection) [38-40]. When validating assessments remotely, the unsupervised and uncontrolled nature of the study environment and the potential for selection bias need to be considered. Table 1 illustrates not only some limitations of remote data collection but also the advantages that remote data collection offers over data collection at testing facilities. The advantages may offset the disadvantages of remote data collection because research suggests that data collected remotely and in-person are comparable [1,39,41-45].

**Table 1:** Key factors to consider when validating assessments for remote research.

Factors	Limitations	Advantages
Absence of rater or supervision	The researcher cannot observe participants to determine whether participants are incapacitated, disengaged, or require clarification and intervene if necessary [46].	Participants may be less influenced by social facilitation or impairment and behave more naturally [47].
No central testing location and testing can occur at unspecified times	There may be a higher likelihood of distractions during data collection [48]. The sample might be biased toward individuals with technology and internet access and technology proficiency [1,39,48,49].	Being outside of the laboratory or clinic may reduce evaluation apprehension and cause participants to behave more naturally [50]. Depending on the study design, participants may be reporting on behaviors, mood states, etc when and where they naturally occur [3]. Participation in the study is accessible to individuals who are unwilling or unable to travel to a central testing location or to be tested in person [1,48].
Differences in device, computer hardware, software, processing speed, screen resolution, display characteristics, internet connection, and response input method	May bias stimulus presentation and response measures, especially reaction time [1,40,45,48,51,52] Differences in the ownership of certain devices (eg, smartphones) may be patterned by sociodemographic factors [53].	Having participants use their personal devices to input data may reduce study costs (devices do not need to be purchased and supplied to participants). In addition, the use of a familiar device may improve performance and compliance [54].

Evaluating behavior under controlled (ie, laboratory or clinic) or quasi-controlled (ie, on the web) conditions may suffer from poor external validity because the findings will not necessarily represent natural behavior. External validity refers to the degree to which the measurements generated by an assessment generalize to other people (population validity) and settings (ecological validity) [35] and across time [55]. In field research, behavior can be evaluated at frequent intervals, in natural settings, and in real time. This avoids experimenter and recall biases, increasing the ecological and temporal validity of the research. The population validity of field research is less straightforward (Textbox 1).

**Taking a closer look at the external validity of remote assessments.**

**Selection biases**

- Both remote and in-person studies are subject to selection biases [49,56-60]. Whether a study is conducted remotely or in-person, participants are motivated to take part for a variety of reasons. For example, a common motivation for taking part in in-person studies is financial reward; other reported motivations include the desire to help science and medicine, help other people, learn, and socialize [60]. Differences in participation motivation can have downstream effects such as affecting engagement with the study procedures. In turn, data quality may suffer, resulting in misleading findings. Previous research demonstrates that there are systematic differences in engagement between paid and course-credit participants in in-person studies [59] and between web-based participants looking for paid work (eg, *Amazon Mechanical Turk* users) and those recruited through paid advertisements [56].
- A large proportion of the participants in in-person studies are Western, educated, affluent, and democratic individuals from industrialized countries [61] and primarily students [1,48]. The resulting lack of diversity of the sample can weaken population validity [1,48,61]. For example, there is evidence of systematic differences in data obtained from student samples and the general population [57]. When conducting research with clinical populations, there are additional barriers associated with poor health that can bias trial recruitment and retention [62]. Individuals with the lowest levels of functioning may be the least likely to participate in, or the first to drop out from, clinical trials because participation might be too burdensome [63]. Conversely, when recruitment for clinical trials primarily occurs at health care facilities, individuals who do not visit doctors’ offices and hospitals (perhaps those with the highest levels of functioning or those that dislike or fear health care settings) are likely to be underrepresented in the clinical research [64]. Collecting data through remote assessments may increase sample diversity, for instance, by making participation more accessible to nonstudent populations (such as individuals who work during normal operating hours, who have care responsibilities, who live and work far from the university, who are unfamiliar with research, etc). Collecting data through remote assessments can also increase sample diversity for clinical research by making participation more accessible to individuals with varying disease severity and to those reluctant to seek out treatment. Remote methodology also allows individuals who might not otherwise participate in research because of disapproval from family or friends [65] to participate discretely.
- However, although this approach mitigates certain selection pressures, it is likely to induce different selection effects relating to, for instance, internet and device access [49,54,66,67]. Although it is commonly accepted that this may affect generalizability, it may also bias exposure-outcome relationships within the study of interest owing to collider bias. For example, say we enroll participants in a remote study on cognitive performance in which assessment necessitates using an iPhone. It has been demonstrated that ownership of an iPhone is associated with educational attainment, age, and health [68]. If we assume that educational attainment is related to cognitive performance, then any relationship we see between the predictors of iPhone use (eg, age and health) and cognition may be distorted by collider bias [69].
- Instead, researchers may allow participants to complete the assessments on any smartphone to increase the inclusivity of the research. If large variations in responses due to software or hardware differences are anticipated, the analysis may include device type as a covariate to control for this variability. Doing so, however, can again introduce collider bias, where, for example, an association between socioeconomic status and cognitive performance may appear weaker than the true population value [54]. This selection bias poses a risk to generalizability of the findings. Therefore, researchers must carefully consider their recruitment strategy and implement statistical tools such as weighted and sensitivity analyses to avoid and correct for selection biases [58,70].

## How to Validate High-Frequency Assessments for Remote Research

Field assessments cannot feasibly be administered in controlled settings at fixed times in an attempt to avoid interference from the outside world. As a result, it can be challenging to empirically evaluate the impact of extraneous factors and thus demonstrate robust internal validity, especially because there can be substantial intraindividual variability in many important symptoms and behaviors. One solution is to exploit the ability of these tools to capture high-resolution data [22]. High intraindividual variability can inflate the sample SD; increasing the number of data points per participant can increase the precision of estimates and improve statistical power [71-74]. However, the feasibility of increased sampling needs to be considered because it can exacerbate practice and fatigue effects [33].

To validate any new high-frequency assessment, the procedure is broadly the same as that for a low-frequency assessment: outcomes from a high-frequency assessment can be compared with those generated by an established low-frequency assessment. As high-frequency assessments are administered under flexible conditions, they exhibit greater external validity at the expense of internal validity. Likewise, because low-frequency assessments are administered under stricter conditions, they exhibit greater internal validity at the expense of external validity (Textbox 1). Therefore, equivalence in the outcomes generated by these complementary measures suggests that high-frequency assessments are likely to possess robust external and internal validity.

However, to make a comparison between two complementary measures, any methodological differences in how the measures are implemented need to be taken into account. When validating an assessment for high-frequency testing, the new assessment is often a brief assessment, whereas the established assessment may be a full-length assessment. When validating a brief assessment against a full-length assessment, there is often a temporal mismatch. A full-length assessment needs to be administered only once to provide meaningful data. However, because a brief assessment is less comprehensive than a full-length assessment, a single data point may be less likely to be informative. Instead, the brief assessment needs to be administered repeatedly across a range of time points, with the resulting multiple data points taken together to provide useful information.

An exception might be when a full-length assessment is not particularly lengthy to begin with; therefore, the abbreviated version is not considerably shorter than the full-length version, and the psychometric properties of the assessment are not drastically affected. The reason for shortening the assessment might be to coadminister it alongside other assessments while keeping the total testing time brief. Alternatively, it might be beneficial to remove a component that may be problematic when delivering assessments remotely. For example, the 8-item PHQ is equivalent to the 9-item PHQ except that it excludes the item on suicidal ideation. It is useful for screening for depression in environments where it would not be feasible to implement safeguarding procedures for participants who indicate suicidal thoughts or intentions [75]. In these cases, the assessment may still be administered at high frequencies, but not because increased sampling is necessary to compensate for a reduction in trials or items. Therefore, in these cases, the outcomes from a single abbreviated assessment would be meaningful and could be directly compared with the outcomes from a single full-length assessment.

When validating a brief assessment against a full-length assessment, there is often a spatial mismatch. Brief assessments are often implemented to facilitate field research, whereas full-length assessments are best suited to more controlled environments where participants can dedicate a substantial amount of time to attend to the assessment (ie, in a clinical or laboratory setting). Therefore, when comparing outcomes between the two it is useful to compare high-frequency measurements collected in the field with low-frequency measurements collected in a controlled environment. For example, a recent study evaluated the feasibility and validity of high-frequency cognitive assessments in patients with schizophrenia. Patients and healthy controls completed a traditional neuropsychological battery at the clinic, followed by an ecological momentary assessment (hosted on a mobile phone) to measure cognitive function remotely for 7 days. Compliance was high, fatigue effects were not observed, and practice effects occurred as a function of study duration, but this relationship was observed for both the patient and control groups. Outcomes for the high-frequency abbreviated assessments correlated considerably with the outcomes from the validated full-length assessments in both patients and controls, demonstrating convergent validity for the high-frequency assessments [76]. However, the full-length assessment, against which the abbreviated assessment is validated, does not necessarily need to be administered at a testing facility. It can instead be administered remotely if it has been validated for remote administration and can feasibly be administered at moderately high frequencies (eg, once a week or once a day). This approach is illustrated in Textbox 2 [77].

**Comparing high-frequency abbreviated assessments with low-frequency full-length assessments.**

**Aim**

- To evaluate the feasibility and validity of high-frequency assessments to capture fluctuations in cognition and mood

**Methods**

- Ecological momentary assessment was used to measure cognition and mood remotely for 2 weeks in 10 healthy participants in a pilot study.
- Cognitive function was assessed remotely by using the following:
  Validated full-length assessments from the Cambridge Neuropsychological Test Automated Battery (CANTAB): spatial working memory, rapid visual information processing, attention switching task, and emotion recognition task. The assessments were hosted on a web page and administered after 5 PM each day.
  An abbreviated assessment (hosted on a smartwatch: Microsoft Band 2) of working memory (A-prime: the ratio of hits [correct detection of an n-back match] to false alarms [response during no match] 2-back task). The assessments were administered once per hour between 9 AM and 7 PM.
- Mood was assessed remotely by the following methods:
  A validated full-length assessment: positive and negative affect schedule. The assessment was hosted on a web page and administered after 5 PM each day.
  A brief assessment (hosted on a smartwatch: Microsoft Band 2) of emotional state (through the selection of the participant’s current emotion and rated intensity of this emotion) probed immediately after cognitive testing each day.

**Key findings**

**Figure 2:**
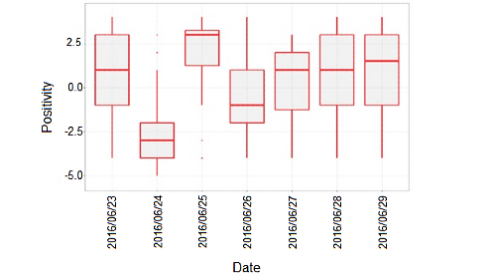
Daily mood positivity across all participants over a 7-day period.

- The feasibility of the high-frequency methodology was evaluated by measuring compliance with data collection. The high-frequency 2-back task was completed on 64% (9/14) of the study days, with an average of 3.6 tests completed on those days. More assessments were completed on weekdays than on weekends and outside of commuting hours (9 AM and 6 PM).
- The convergent validity of the high-frequency 2-back task was evaluated by correlating its outcomes with the outcomes from the CANTAB tests. A-prime was significantly correlated with measures of spatial working memory (*r*=−0.8) and attention switching task (*r*=−0.45), and moderate but not statistically significant correlations were observed with performance on a measure of sustained attention (rapid visual information processing A-prime *r*=−0.33). On the high-frequency assessments of mood, this nonclinical sample rated the mood as generally positive and of a low intensity. Participants were first asked to rate their emotion by choosing 1 of 6 canonical emotions (happiness, sadness, disgust, fear, surprise, and anger) and then the intensity of that emotion on a 6-point scale where 6 was the most intense. As negative emotions (sadness, disgust, fear, and anger) were much less frequently reported than positive emotions (happiness and surprise) in this healthy sample, daily intensity reports across positive and negative emotions were aggregated to produce a single scale representing the overall balance of reports of positive or negative emotional intensities over a day. Notably, a reduction in mood positivity was observed on the day of the results of the 2016 United Kingdom European Union membership referendum (June 24, 2016; Figure 2).

**Key conclusions**

- A full-length assessment allows for comprehensive data collection at a single time point and allows for researchers to exert greater control over the testing environment. Yet, the data might be distorted owing to low-sampling frequency or use of an artificial environment. In this case study, it was feasible to administer the full-length assessments daily in natural settings. However, many full-length assessments might be too long to administer as frequently as once a day [78] or need to be administered at a testing facility [76] (eg, when specialist equipment or a trained administrator is required). The results in this case study demonstrate how extraneous factors (eg, the referendum) can affect outcomes. Outcomes that are measured infrequently are more vulnerable to confounding bias (ie, the outcomes may differ dramatically depending on the day or time when they were measured). Measuring outcomes at high frequencies, instead, allows researchers to detect confounding effects and control for, or investigate, them as appropriate.
- To feasibly measure outcomes at high frequencies, assessments must be brief, which means the comprehensiveness of data collected at a single time point is drastically reduced. Therefore, to ensure that abbreviated assessments are sensitive to what they are intended to measure, they need to be administered more frequently than, say, once per week or once per day. This often requires sampling to occur in real-world environments because it is impractical to collect data in a laboratory or clinic at such high frequencies. Using field assessments comes with its own set of challenges; therefore, study designs should account for times when engagement may be low (eg, weekends or during commuting).
- Although it is unfeasible to validate field assessments under tightly controlled conditions, they can be compared with assessments that have reliably exhibited internal validity in both healthy participants and patient groups, such as the CANTAB tests portrayed in this case study [79-83]. Field assessments benefit from sampling phenomena within natural settings and in real time over extended periods of time. When outcomes generated by the field assessments are comparable with those generated by validated full-length assessments, it is reasonable to assume that the field assessments also exhibit strong internal validity or, at the very least, are sufficiently valid measures owing to robust external validity and extensive sampling.

One disadvantage of validating a high-frequency assessment against a low-frequency assessment is that it seeks equivalence between outcomes captured at different times and in different locations. An alternative approach is to compare 2 assessments that take measurements in equivalent ways (ie, at high frequencies in the field). This approach is only possible when a brief assessment (validated to measure the same or an empirically similar construct of interest) already exists. This approach allows for a new brief assessment to be validated against an established assessment in real time, evaluating construct and criterion validity. As the assessments are completed concurrently, both assessments will be subject to similar influences (eg, common confounding structures). However, this threat to internal validity is likely to be offset by the richer and more granular data produced by high-frequency assessments. It allows for in-depth exploration of interindividual and intraindividual variability, which is key to identifying a signal in a noisy setting. This validation approach is illustrated in Textbox 3. Although one of the strengths of high-frequency testing is increased external validity (Textbox 1), it is worth thinking critically about the degree to which it increases generalizability (population validity) specifically, with special consideration given to recruitment strategy and analytical approaches [58,70].

**Comparing different high-frequency assessments.**

**Aim**

- To evaluate the feasibility and validity of a high-frequency assessment of vigilant attention and explore how reducing task length affects both factors

**Methods**

- Ecological momentary assessment was used to measure vigilant attention remotely for 2 weeks in 13 healthy participants in a pilot study.
- Vigilant attention (which is sensitive to sleep deprivation) was assessed remotely by using the following:
  An abbreviated version of the psychomotor vigilant task (PVT), an objective measure of vigilant attention. The PVT measured reaction time after stimulus onset across approximately 50 trials. It was hosted on a mobile phone and was administered up to 2 times per day (morning and afternoon).
  A subjective, validated measure of sleepiness or alertness, the Karolinska Sleepiness Scale (KSS). The KSS consists of a single rating of sleepiness or alertness on a 9-point scale. The KSS (hosted on a mobile phone) was administered immediately after each administration of the PVT.

**Key findings**

**Figure 3:**
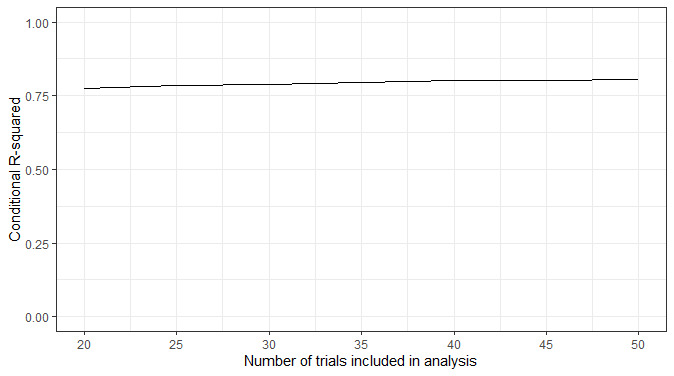
The proportion of variance explained by the model (conditional *R^2^*) across all participants based on the number of trials that were included in the analysis.

**Figure 4:**
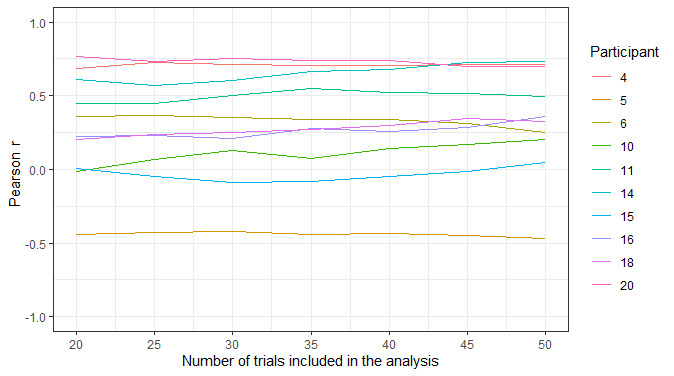
The correlation (Pearson *r*) between psychomotor vigilance task reaction times and Karolinska Sleepiness Scale scores across all time points within participants. Only participants with more than 50% compliance (ie, completed at least 14 of the 28 possible assessments) are included (n=10).

**Figure 5:**
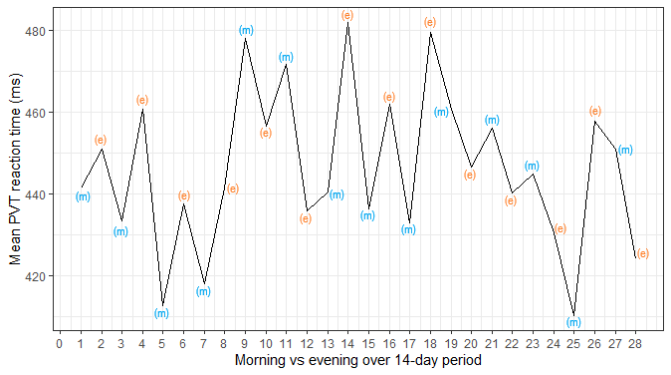
Mean psychomotor vigilance task reaction time (across all participants) as a function of the time of day (morning vs evening) and study day (day 1 to day 14). PVT: psychomotor vigilance task.

- Compliance was poor; of the 13 participants who took part, 10 completed at least 50% (14/28) of the assessments. However, it should be noted that the PVT and KSS measures were administered as part of a longer battery (approximately 10 min), which many of the participants felt was burdensome. Therefore, it is probable that compliance would have been higher if the PVT and KSS measures were administered on their own. Overall, mean PVT reaction times were correlated moderately with KSS scores (*r*=0.37; 95% CI 0.25-0.48). As the outcome measures were captured in real time and sampled frequently, they were not influenced by retrospective recall bias and were less susceptible to coincidental factors. In addition, the granularity of the data allowed for in-depth analysis of how compliance, task performance, and task sensitivity changed as a result of repeated assessments, the time of day, the day of the week, task length, and individual differences.
- For example, to assess if the PVT’s sensitivity to sleep deprivation changed as a result of task length, the association between PVT reaction times and KSS scores across all time points for the full 50 trials can be compared with the first 45, 40, and so on, trials. As there are multiple observations for each individual, a mixed model can be used to account for the dependency of observations, where observations (level 1) are nested within participants (level 2). Below, we have plotted the proportion of variance explained by the model based on the number of trials included in the analysis (Figure 3).
- The plot illustrates that the amount of variation in PVT reaction time explained by KSS scores does not change when fewer trials are included in the analysis. However, this plot depicts aggregate data across all participants. Owing to the granular nature of the data, several data points exist for each measure for each participant. Therefore, it is possible to calculate an intraindividual correlation between 2 variables (such as PVT reaction times and KSS scores) for each participant to allow for the interpretation of interindividual and intraindividual variability. Below, we show the association between PVT reaction times and KSS scores across all time points for each participant with more than 50% (14/28) compliance (n=10) based on the number of trials included in the analysis (Figure 4).
- In addition, owing to the granular nature of the data, sources of variability such as temporal influences on PVT performance (eg, time of day, repetition of assessments) can be explored and accounted for in the modeling. Below, we show how mean PVT reaction time (averaged across all participants) varies based on time of day (morning vs evening assessments) and over the 14-day testing period (Figure 5).

**Key conclusions**

- The rich, granular data produced by the high-frequency assessments allow for in-depth exploration of interindividual and intraindividual variability. Furthermore, sources of random variability can be accounted for in the analysis, increasing the signal-to-noise ratio.

Longitudinal data sets, such as those presented in Textbox 2 and Textbox 3, can be analyzed using mixed-effect models, which allow for both fixed and random effects to be included in the modeling. The benefits of using mixed-effects models are that they can tolerate missing data and evaluate changes over time. Furthermore, changes over time can be explored with respect to how each individual changes over time and how this differs among individuals [84,85]. New approaches to mixed-effects modeling are being developed that allow for close investigation of within-individual volatility. This approach is illustrated in Textbox 4.

**Deriving novel phenotypes using fine-grained repeated observations.**

**Measuring response volatility**

**Figure 6:**
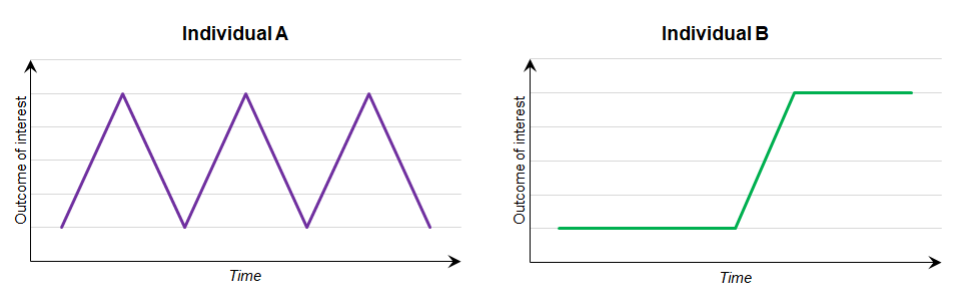
Within-individual repeated observations of an outcome of interest with identical means and SDs but different volatility.

- Fine-grained temporal data such as those offered by the more rapidly reflexive nature of remote research assessments allow researchers to test hypotheses that could not be tested with coarser temporal coverage. For instance, ecological momentary assessment studies can be deployed more rapidly than traditional surveys owing to their electronic distribution—meaning they also allow researchers to ask more reflexive questions, for instance, about the mental health impact of rapidly evolving events such as COVID-19 lockdown policies [86].
- There are further benefits to the use of remote research assessments in the generation of higher-order individual-level characteristics. Repeated measures collected from an individual over time allow for inference about not only the nature of a static response characteristic, but also the within-individual heterogeneity within the response of interest. This is clearly of particular interest in psychological research if it is hypothesized that the variability itself is of substantive interest. For instance, work on borderline personality disorder can require the characterization of affective dysregulation through response volatility [87]. Similarly, studies have investigated associations of affective volatility with mental health and alcohol consumption mediated by mean positive affect in mothers [88].
- Broadly, further increases in temporal granularity allow more complex parameterizations of volatility. We may start with something relatively commonplace such as SD or variance. However, consider the example below: intuitively we can see that individuals A and B have different levels of *volatility*; yet, simply considering the SD or variance of measures will yield identical values for both participants (Figure 6).
- If the researcher wants to distinguish between these individuals, then variance or SD is clearly insufficient. We must include further consideration of, say, autocorrelation or stability [89], or even something more bespoke. For instance, researchers monitoring continuous blood glucose levels from wearable technologies derived a measure of “variability from one moment to the next,” operationalized as the length of the line on a graph between 2 adjacent time points [90].
- The fine-grained data afforded to researchers by remote research assessments allow the generation of more complex research questions. For example, take the data presented in Textbox 3. Novel mixed-effect models could be specified to further explore the association between sleep quality and alertness. This would allow for analysis of not only whether sleep quality informs mean levels of alertness, but also whether the variability of the responses of a given individual are predicted by their indicated sleep quality.

**Key conclusions**

- Fine grained, repeated temporal measures allow researchers to derive novel phenotypes from repeated observations of a given outcome. Extracting and modelling higher order observational phenomena will, in turn, enable better understanding of underlying, within-individual processes underpinning effects in traditional observational enquiry.

## Conclusions

Remote research assessments can be used to study cognition and behavior in unconventional and innovative ways while carefully adhering to established research principles. As a result, the use and further development of these assessments will reshape psychological and clinical research in the near future. These tools are not without their own set of unique challenges and require the careful consideration of the optimal approach, particularly approaches for increasing generalizability, for any given research question. This presents an opportunity for discoveries that, without creative thinking, technological advancements, and flexibility, might otherwise have remained undiscovered. There is always room to improve research tools, and it is vital that the methods to evaluate these tools keep pace.

## Abbreviations

PHQ: Patient Health Questionnaire
